# Hospital Sunday Amenities

**Published:** 1895-02-09

**Authors:** 


### The Institutional Workshop.

### HOSPITAL ADMINISTRATION.

### HOSPITAL SUNDAY AMENITIES.

It will be remembered that in our issue of December 15th, 1894, we called attention to the fact that the largest and richest congregation of Coventry, that of St. Michael's, had given no collection to the hospital for three years past. The Rev. Dr. Mills, the vicar of St. Michael's, stated that three years ago he was compelled to withdraw his annual subscription, and to refuse to include the hospital amongst the charities which he put before his congregation at that time, and within the last few months he has put in writing his reasons, at the request of one of the officials. Dr. Mills declared that these reasons are known to the committee and are unanswerable. Since we published this important accusation against the Coventry and Warwickshire Hospital and those connected with its management, no notice appears to have been taken either of the Rev. Dr. Mills' statement or of our comments thereon. We beg, therefore, once more to call upon the authorities of the Coventry and Warwickshire Hospital for some reply or explanation, failing which it would seem probable that the governors of the institution may find it necessary to call a special meeting in order to demand an explanation at the hands of the committee. Either the Rev. Dr. Mills has exaggerated the importance of his reasons, or the authorities of the Coventry and Warwickshire Hospital are greatly to blame for not dealing with them in a practical manner.

